# Impact of Carcass Detection Delays on the Sustained Transmission of African Swine Fever Among Wild Boars

**DOI:** 10.1155/tbed/9889895

**Published:** 2025-07-28

**Authors:** Dae Sung Yoo, Ho-Seong Cho, Yeonsu Oh

**Affiliations:** ^1^Department of Preventive Veterinary Medicine, College of Veterinary Medicine, Chonnam National University, Gwangju 61186, Republic of Korea; ^2^College of Veterinary Medicine and Bio-Safety Research Institute, Jeonbuk National University, Iksan 54596, Republic of Korea; ^3^Department of Veterinary Pathology, College of Veterinary Medicine and Institute of Veterinary Science, Kangwon National University, Chuncheon 24341, Republic of Korea

**Keywords:** African swine fever (ASF), carcass decomposition, detection delays, postmortem interval, spatiotemporal patterns, suidae family, surveillance, wild boar

## Abstract

African swine fever (ASF) is a devastating infectious disease caused by the ASF virus (ASFV), a member of the *Asfaviridae* family, which primarily affects species within the Suidae family, including several African wild boars, such as the warthog (*Phacochoerus Africanus*). ASFV is characterized by its robust double-stranded DNA genome and is transmitted through various transmission routes, including direct contact with infected pigs or fomites, ingestion of contaminated swill, and ticks from the *Ornithodoros* genus. Upon entry, the virus induces cell apoptosis, systemic hemorrhage, and high fever, typically leading to a near 100% fatality rate among affected pigs, thereby causing substantial losses to global swine populations. Similar to ASF outbreaks in other countries, South Korea reported its initial ASF infections in domestic pig farms in September 2019, following an incursion in wild boar populations. Subsequently, the virus propagated southward, tracing the natural migratory paths of wild boars through forested and mountainous regions, and sporadically infecting nearby pig farms. In response, robust surveillance of wild boar populations has become crucial, as these efforts provide timely and essential information to stakeholders. Effective and prompt removal of ASF-infected carcasses is critical, as these carcasses can remain infectious for extended periods, thereby posing a continuous risk of secondary outbreaks. This study conducts a comprehensive analysis of the spatiotemporal patterns of ASF-infected wild boar carcasses, based on 41,192 wild boar samples collected through active and passive surveillance from September 2019 to March 2022. It examines how environmental conditions, such as forest density, regional humidity, and geographical terrain impact carcass decomposition rates and consequently, the detection timelines of ASF-infected wild boars. This research aims to pinpoint factors contributing to detection delays and refine strategies for early detection and rapid removal of contaminated carcasses, thereby enhancing control measures and mitigation efforts against ASF in affected regions.

## 1. Introduction

African swine fever (ASF) is a lethal infectious disease caused by a virus belonging to the genus *Asfivirus* within the *Asfaviridae* family, characterized by a large double-stranded DNA structure. The susceptibility to this virus is confined to species within Suidae family, with several African wild boar species, such as the warthog (*Phacochoerus Africanus*), serving as natural reservoirs. The disease spreads through direct contact with infected pigs, consumption of contaminated swill, contact with fomites, and vectors, such as soft ticks from *Ornithodoros* genus, which infect and cause apoptosis in immune cells leading to systemic hemorrhaging and high fever, often resulting in fatality. Studies indicate that ASF-infected pigs have an almost 100% fatality rate, causing significant swine population losses globally [1].

Once introduced into a disease-free country, the ASF virus (ASFV) can circulate among both wild boars (*Sus Scrofa*) and domestic pigs (*Sus Scrofa domesticus*), complicating eradication efforts due to the geographical and biological connections between these populations. For example, Poland has experienced ongoing incidences of ASF since its first detection in wild boars in 2014, propelled by factors like domestic pig production chains and the life cycle of wild boars [2].

Similarly, South Korea witnessed its first cases of ASF in domestic pig farms in September 2019, spatiotemporally close to the virus was detected in wild boar populations. In South Korea, all ASF cases identified since the 2019 outbreak have been attributed to genotype II strains, closely related to the highly virulent Georgia 2007/1 strain that has been responsible for widespread outbreaks across Europe and Asia [3]. The ASFV has been spreading gradually southward, following paths through forests and mountainous areas preferred by wild boars, occasionally infecting nearby pig farms [4, 5]. Its southward spread pattern aligned with natural wild boar movement corridors, resulting in sustained viral presence in affected landscapes [6]. Additionally, the basic reproduction number for ASF among wild boar populations in South Korea has been estimated between 1.11 and 2.37, indicating a significant potential for spread [7]. Moreover, the basic reproduction number gradually increased year after year mainly due to the viral propagation into the dense region of wild boars, raising the concern over the incursion of the virus into domestic pig farms [8]. Although precise density data are lacking, habitat suitability models suggest that the affected northeastern regions of South Korea support relatively high wild boar densities, especially in forested and mountainous areas. A previous study [9] has used species distribution modeling to estimate relative abundance, which informed our use of habitat suitability as a proxy in this study.

The high rate of ASF notifications in South Korea's wild boar population, particularly noted for its proactive surveillance measures, reflects an intense effort to manage the disease's spread [10]. For instance, from October, 2019 to March 2021, over 41,187 samples were collected from wild boars, with carcasses showing a positivity rate of approximately 26%, markedly higher than other sample types which showed less than 1% positivity [3, 11].

Given the critical role of wild boar surveillance, it is essential to provide timely information for stakeholders, such as early warning and risk areas [12]. Furthermore, since the carcasses are likely to remain infectious for an extended period, the rapid and timely removal of ASF-infected carcasses is a very critical action to minimize the local persistence of the virus, a potential source of the secondary outbreak [13]. This study focuses on the persistence of the ASFV in carcasses that can potentially lead to secondary outbreaks. The rapid and effective removal of ASF-infected carcasses, therefore, is imperative. South Korea's animal health authorities have actively intervened within a 10 km radius of localities where ASF is detected in wild boars, aiming to reduce the virus's local persistence. However, challenges persist due to biases in the ASF surveillance program's focus primarily on the carcass search, which does not adequately consider the postmortem interval (PMI) and time of capture, potentially leading to delayed detections [6, 13]. By analyzing the spatiotemporal patterns of carcass detection and the factors influencing the timeliness of these detections, this research identifies significant contributors to these delays using a Bayesian framework. The findings are intended to enhance the strategies for early detection and prompt removal of contaminated carcasses, thereby mitigating the risk of further spread and impact of ASF.

## 2. Materials and Methods

### 2.1. ASF Surveillance Data in Wild Boar

In South Korea, from 1 September 2019 to 31 March 2022, a total of 41,192 samples were collected from wild boars through active surveillance (28,330 wild boars by hunting, 5441 wild boars by trapping) and passive surveillance (7421 wild boars found dead by active search or voluntary reports). Out of these samples, 2464 tested positive for ASF, with the initial detection confirmed on 2 October 2019. Notably, 89.8% (2212 out of 2464) of the positive samples came from wild boar carcasses.

Carcass searches were conducted by multiple patrol teams designated to the 16 municipalities where ASF infections in wild boars or nearby areas were identified. In other regions, carcass detections were primarily through voluntary reports by the public and designated hunters. The ASF surveillance data included the type of sampling method (i.e., carcass, hunting, and trapping), sampling date (i.e., detection date), geographical coordinates, and PMI estimates if the sample tested positive and was collected from a carcass. Based on experimental and field data from genotype II infections that circulated in South Korea, wild boars typically succumb within 4–10 days postinfection, depending on viral dose and individual susceptibility [8]. This short clinical course supports the use of PMI as a proxy for detection delay and environmental persistence. This data was provided by the Ministry of Environment, South Korea.

### 2.2. Study Area

Surveillance records indicate that the wild boar population in the northeastern region of South Korea, covering approximately 11,106 km^2^, has been affected by ASF outbreaks. Therefore, our study focused on these ASF-affected areas to investigate the variance of PMI as an indicator of delayed detection and the infected periods (IPs). PMI is used as a proxy for delayed detection that might indicate a higher risk of viral contamination and transmission among the wild boar population. As PMI represents the time interval from the death of an ASF-infected wild boar to the detection date normally followed by removal from the environment, the longer PMI estimates could suggest a higher possibility of the ASFV being persistent and transferred among the population.

To accurately estimate the PMI and IP across the designated surveillance area, we partitioned all Korean territory using a hexagon-shaped grid with a size of about 28.26 km^2^, equivalent to the circle with a 3 km radius. This grid size was chosen based on the home ranges of wild boars in South Korea as identified in prior studies [6]. Each grid was then analyzed for the presence of ASF-positive wild boar carcasses, and subsequent calculations for PMI and IP were performed only on those girds that contained ASF-positive detections. This grid-based approach allows for a more precise spatial analysis of infection spread and carcass persistence. For a detailed explanation of the spatial partitioning and the methodologies used for calculating PMI and IP, see S1, which outlines the initial assumptions and the subsequent analytical procedures employed.

### 2.3. Environmental and Anthropogenic Variables

Seven environmental and anthropogenic variables, summarized in Table S1 in the Supporting Information, were used to investigate their relationship with area-specific PMI and IP. These variables were selected based on existing literature identifying key factors influencing both the detection of ASF-infected wild boars [13–15], and the risk factors associated with ASF infection in these populations [16, 17].

Environmental variables included the proportion of forest, water bodies, and rice paddies within each hexagonal grid area (~28.26 km^2^). These proportions were derived from a 5-m resolution land-use map. Road density, defined as the proportion of land area covered by roads, was calculated using road data obtained from the Ministry of the Interior and Safety, South Korea, in 2018.

In addition to the proportions of land use, environmental variables for each area were employed using raster data, represented as mean values. These included the normalized difference vegetation index (NDVI), heat load index (HLI), and topographic wetness index (TWI). These indices were calculated using elevation data from the United States Geological Survey's Global Digital Elevation Model, derived from the Shuttle Radar Topography Mission, conducted in 2014 at a spatial resolution of 1 km. The TWI, an indicator of water accumulation and local drainage, was calculated as ln (α/tanß), where α represents the total catchment area per flow width and (i.e., a cumulative upslope area supplying water), and tanß represents the slope gradient between upstream and downstream cells. This index serves as a proxy for soil moisture, integrating both upslope water supply and downslope drainage patterns.

To assess carcass search and reporting accessibility by the general population, average human density data (worldpop.org, 2019) was used to calculate the average cost distance of each area from residential areas, using elevation as the cost factor.

Surveillance intensity, defined as the total number of wild boar samples collected within each area from the start of the predefined risk period until the first detection of ASF-positive carcasses, was also included. This risk period commenced 1 week after the first reported ASF infection in neighboring areas (defined as grids sharing a border or point, using queen contiguity), based on a transmission rate of 3 km per week [5]. Sensitivity analyses extended this period to 2 and 3 weeks post initial infection in neighboring areas.

The geographical distribution of ASFV infection in wild boars is hypothesized to correlate with actual wild boar distribution. Due to the lack of direct wild boar density data for South Korea, habitat suitability was used as a proxy, following a study by Kim and Pak S.I [9].

Given the potential for spatial autocorrelation among environmental and anthropogenic variables, a Global Moran's *I* test was conducted to assess this phenomenon. The null hypothesis posited the absence of spatial autocorrelation among the study variables.

### 2.4. The Identification of Impact of Delayed Detection of ASF-positive Carcass on the Infected Period (IP)

We hypothesized that the delayed detection of ASF-infected carcasses could contribute to extending the IP of ASF within the wild boar population. To test this hypothesis, we initially estimated the correlation between area-specific PMI and IP using the Clifford–Richardson modified test, which is a corrected Pearson's correlation test for spatial autocorrelation. This test adjusts the actual sample size of a test statistic to an effective sample size, accounting for changes in the variance of two data values due to spatial autocorrelation [18].

To further understand the local effects of delayed detection on extending ASF infection periods in neighboring areas, we employed the bivariate local Moran's *I* statistic. This analysis was crucial for identifying spatial clusters of high PMI and high IP, indicating areas where delays in detection might significantly influence the spread of ASF. Details of this analysis, including the definition of the statistical parameters and the interpretation of results, are elaborated in S2. Additionally, to robustly model the association between PMI and IP, we built a Bayesian gamma regression model. This model adjusted for covariates, such as habitat suitability for wild boars and surveillance intensity, incorporating a spatial random effect term using an intrinsic conditional autoregressive (iCAR) function to account for spatial autocorrelation of residuals. The methodology, the detailed setup of this Bayesian model, and the interpretation of its results are provided in S2.

The SpatialPack version 0.4 software package in R version 4.2.1 was employed to conduct the Clifford–Richardson modified test [19]. This comprehensive approach ensures a deeper understanding of how delayed detection impacts ASF transmission dynamics within wild boar populations.

### 2.5. Identification of Environmental and Anthropologic Contributors to Delayed Detection of ASF-Positive Carcass

Area-specific PMI, representing the time elapsed between the death of an ASF-infected wild boar and its subsequent detection and removal, can be analyzed using a time-to-event statistical regression model. Therefore, area-specific PMI was modeled as a survival curve. A survival analysis framework, employing a Cox proportional hazards model with a frailty term that accounts for the intrinsic conditional autocorrelation, serving as a random effect to adjust for the observed spatial autocorrelation of area-specific PMI as detailed in a prior study [20].

To further elucidate the factors influencing the timely detection and removal of carcasses, we conducted a Bayesian Cox regression analysis within this survival framework. This model assesses the hazard at any given time, which is conceptualized as the probability of an ASF-infected carcass being removed at that time. The hazard function, incorporating model parameters that represent the instantaneous rate of carcass removal, is exponentially related to environmental conditions and modeled using a spatially continuous latent Gaussian field.

The complete methodology for estimating the hazard function, the selection and transformation of explanatory variables into categorical variables for model fit, and the verification of proportional hazard assumptions using scaled Schoenfield residuals are all thoroughly detailed in S3. Additionally, the Bayesian framework employed for the model, involving Markov chain Monte Carlo (MCMC) sampling for parameter estimation, is explained comprehensively in this Supporting Information document.

The detailed approach allows for a robust analysis of the environmental and anthropologic factors that significantly affect the detection and management of ASF-infected carcasses, providing insights into optimizing surveillance and intervention strategies. Further details on the Cox regression analysis, including the statistical tools and software used, are provided in S3.

## 3. Results

### 3.1. Overview of an Areas-Specific PMI and IP in ASF-Affected Areas

**Figure 1:**
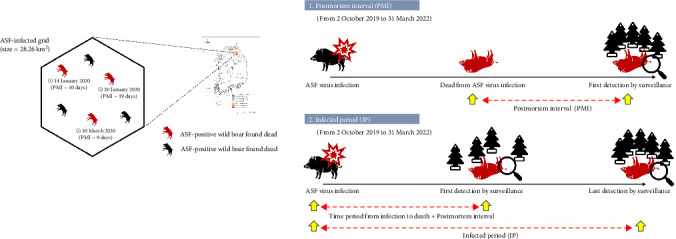
Schematic diagram for definition of area-specific postmortem interval (PMI) and infected period (IP). The date below the ASF-positive wild boar found dead, which was denoted by a red color, represents the detection date. The PMI of ASF-positive carcass labeled by number 1 (10 days) was represented as an area-specific PMI because the carcass was first detected in the given grid. For IP for the given area, 70 days was estimated to be IP as the first infection date was estimated to be January 20, 2020 (minimum of [detection date–PMI–12 days, carcass number 1 = January 23, 2020, carcass number 2 = January 20, 2020, carcass number 3 = March 9, 2020]) and the last detection date of ASF-positive carcass was equal to March 30, 2020.

Table 1 summarizes the statistical distribution of area-specific PMI and IP as a result of the ASF surveillance activities for wild boars from 1 September 2019 to 31 March 2022. in South Korea. Since 2 October 2019, when the first ASF-positive wild boar was detected in South Korea, the ASF-positive carcass was found at 393 out of 5398 grids across the country as displayed in Figure 1.

**Figure 2:**
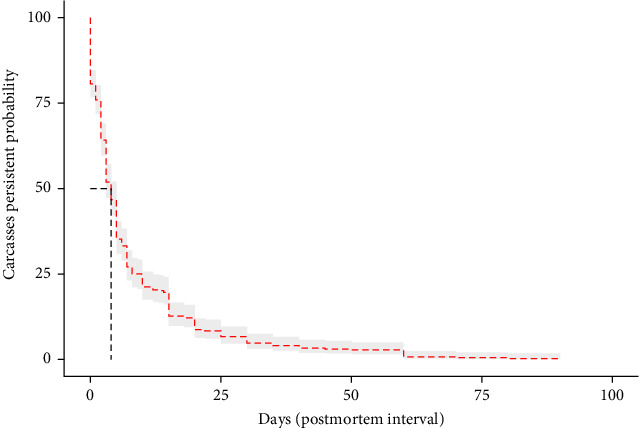
Kaplan–Meier survival curve for an area-specific postmortem interval (PMI) of African swine fever-positive wild boar found dead in South Korea. Gray shading represents 95% confidence interval of survival curve. Vertical and horizontal dotted lines denote the median value of area-specific PMI.

**Figure 3:**
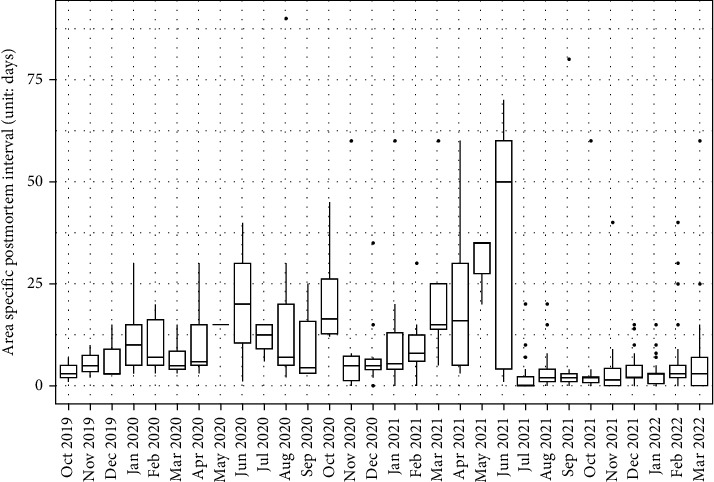
Monthly distribution of area-specific postmortem interval of African swine fever-positive wild boar found dead in South Korea from October 2, 2019 to March 31, 2022.

The average area-specific PMI across ASF-positive grids was 8.25 days, with a standard deviation of 12.08 days. This statistic indicates there is a variability in the time taken to detect carcasses. The median PMI was 4 days, ranging from 0 to 90 days as visually represented by the survival curve in Figure 2. The IP averaged 105.96 days, with a standard deviation of 124.49 days. The coefficients of variation and Moran' I value in the table highlight a moderate spatial autocorrelation, suggesting that neighboring areas tend to exhibit similar PMI and IP values. Additionally, PMI estimates of ASF-positive carcass that were first found at each grid were higher from March (mean of PMI = 26.25 days) to June (mean of PMI = 30.75 days) as shown in Figure 3.

### 3.2. Impact of Delayed Detection of ASF-Infected Carcass on IP of ASF-Affected Areas

**Figure 4:**
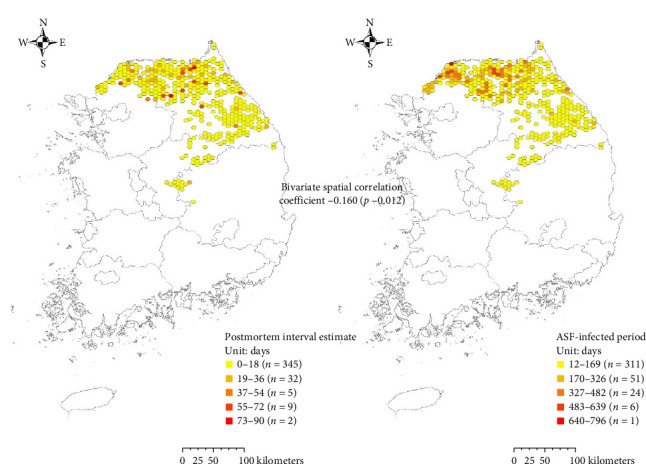
Geographical distribution of surveillance of wild boar population for African swine fever (ASF) and the number of ASF-positive carcass.

**Figure 5:**
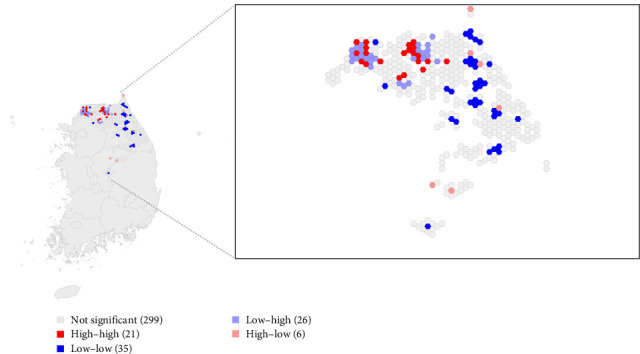
Spatial relationship between area-specific postmortem interval (PMI) and infected period (IP) of ASF in its neighboring areas. The enlarged figure on the right displays the spatial distribution of bivariate local Moran's *I* in which the dark red color denotes the area with high PMI and high IP in its neighbors.

We found a significant positive correlation between the area-specific PMI and the IP, with a correlation coefficient of 0.160 with a *p*-value of 0.012 as illustrated in Figure 4. Additionally, a noticeable local dispersal effect was identified where longer PMIs in specific areas contributed to prolonged IPs in adjacent northern regions, as depicted in Figure 5. 21 clusters were identified where a high PMI was spatially associated with a high IP in neighboring areas. These hotspots indicate that delays in carcass detection in one area are linked to prolonged disease circulation nearby. Furthermore, as summarized in Table 2, the Bayesian gamma regression analysis, which adjusted for surveillance intensity and habitat suitability for wild boars, confirmed a statistically significant positive relationship between area-specific PMI and IP, with a coefficient of 1.01 (95% credible intervals [Crls] = 1.00–1.02).

### 3.3. Environmental and Anthropologic Contributors to Delayed Detection of ASF-Infected Wild Boar's Carcass

Table 3 shows how much environmental and anthropologic factors influence the PMI of ASF-infected wild boars through hazard ratios. Notably, low road density significantly extends detection times, with a hazard ratio of 0.11, indicating that poor infrastructure critically hinders timely carcass recovery. Similarly, dense tree cover showed a hazard ratio of 0.61, moderately contributed to delays detection. In contrast, proximity to water bodies accelerates carcass discovery, evidenced by a hazard ratio of 4.50, Additionally, factors like rice fields and greater distances from populated areas slightly increase the PMI, with hazard ratios of 0.78 and 0.51, respectively.

## 4. Discussion

Many ASF-affected countries continue to report outbreaks in domestic pig holdings, which may result from a combination of factors, including delayed detection and reporting of cases, as well as spillover from ASF-endemic wild boar populations in certain high-risk regions. This situation imposes a significant economic burden on pig producers and compels animal health authorities to invest in extensive prevention and preparedness programs. In this study, we demonstrated that delayed detection of ASF-infected carcass contributes to prolonged virus circulation among wild boars, thereby increasing the risk of transmission to domestic pigs through multiple environmental pathways.

First, our findings highlight substantial variability in carcass detection times (PMI), with some cases exceeding 90 days. The positive correlation between area-specific PMI and the IP suggests that longer detection delays may allow ASFV to persist longer in the environment, prolonging local transmission cycles. This relationship remained significant after adjusting for surveillance intensity and habitat suitability, underscoring the importance of timely detection and removal of carcasses as a potential mitigation measure in areas with ongoing wild boar circulation. We acknowledge that in some ASF-affected regions, the disease has subsided without active carcass removal, likely due to self-limiting dynamics driven by drastic declines in wild boar density. However, in the context of South Korea-characterized by rugged terrain, moderate climate, and continuous viral incursion pressure near the demilitarized zone-ASF has persisted since 2019 despite sustained surveillance. These conditions suggest that self-limitation alone may not be sufficient to interrupt transmission in this setting. Thus, carcass removal should be viewed as a context-dependent control tool rather than a universal solution.

Second, our study elucidates how environmental and anthropologic factors significantly influence the detection times of ASF-infected carcasses in South Korea, where profound economic and logistical challenges have been imposed by ASF. For example, dense forest cover and poor road access significantly delay carcass recovery, while proximity to water bodies facilitates faster detection. These insights can guide targeted surveillance strategies and improve carcass removal efficiency in high-risk areas These natural barriers create obstacles for timely carcass recovery, as dense foliage obscures carcasses, reducing visibility and accessibility. This aligns with seasonal variations, where during the lush vegetation periods of spring and early summer, carcass detection becomes notably more challenging, potentially delaying responses, and prolonging the presence of the virus in the environment.

However, increased human activity in these regions during warmer months seems to inadvertently aid in detection, possibly due to more frequent recreational use of these areas or increased agricultural activity. This observation underscores the complex interaction between human behavior and disease surveillance dynamics.

Similarly, inadequate road infrastructure and poor road conditions hinder access to affected areas, critically allowing carcasses to remain in the environments longer, potentially serving as transmission pathways among the wild boar population. Conversely, proximity to water bodies often correlates with reduced PMIs, as these locations typically provide clearer areas and easier access, significantly aiding surveillance efforts.

Factors like rice fields and greater distances from populated areas slightly increase the PMI, underscoring the impact of terrain navigability and location remoteness on surveillance processes. This highlights the need for adaptive strategies in areas with challenging geographical features to manage and control ASF spread effectively.

Importantly, basic biosecurity practices on pig farms significantly reduce the risk of ASFV introduction, independent of carcass removal efforts [11]. Biosecurity remains the most critical line of defense in domestic swine protection. However, given the persistent environmental reservoir represented by undetected carcasses, particularly in regions with frequent wildlife–livestock interface, carcass removal may serve as a complementary measure to reduce viral pressure on surrounding farms [13].

Drawing on the Belgium experience, the creation of restriction zones and organized, efficient carcass removal strategies were pivotal in controlling ASF spread. Belgium's proactive measures, as documented in their national response, offer a model that could be adapted to suit the geographic and ecological nuances of South Korea [21]. Furthermore, studies like those conducted in Italy have highlighted the effectiveness of various surveillance methods, with passive surveillance proving particularly advantageous in the early stages of outbreak detection [12]. These insights could inform improvements in South Korean strategies, especially in enhancing the effectiveness of early detection systems.

Understanding the dynamics of ASF spread through comprehensive studies like ours provides critical insights that can help refine current strategies and develop more targeted interventions. As ASF continues to pose a significant threat to both wild and domestic swine populations globally, international collaboration and knowledge exchange become paramount. This includes sharing successful containment and eradication strategies, as well as fostering a collaborative approach to research and policy development to mitigate the impacts of ASF.

This study acknowledges several limitations that may impact the interpretation of findings. First, the uncertainty associated with the estimates of the PMI is notable. The PMI estimates are inherently influenced by a variety of environmental and ecological conditions, which adds a layer of complexity to their accuracy. To mitigate this variability, the PMI was determined through methodologies developed by the corresponding institutions' own experiments. Additionally, an expert was consulted to define the interval, aiming to minimize inconsistencies that might arise from differing observations, as communicated personally by the expert involved.

Second, the progression of carcass decomposition is subject to geographical variations, particularly those influenced by local humidity levels. Such environmental factors can significantly alter the rate of decomposition, thus affecting the reliability of the PMI calculations and the subsequent detection timelines of ASF-infected carcasses.

Lastly, the density of the wild boar population is a crucial factor in determining the efficacy of reporting delays. Areas with higher densities of wild boars typically facilitate easier detection and retrieval of carcasses due to the increased likelihood of encountering deceased animals. However, in regions affected by ASF, the variation in wild boar density is not markedly significant, which may limit the generalizability of findings across different geographical settings with varying wild boar populations. Our study did not conduct a cost-benefit assessment (e.g., partial budget analysis), it aimed to underscore how a longer PMI of ASF-infected carcass as could increase the environmental persistence of the virus. Evaluating the feasibility of reducing PMI would be valuable in guiding practical disease control strategies [22]. Thus, the potential utility of such analyses is worthwhile in future work aimed at reducing the PMI.

## 5. Conclusion

In conclusion, our study provides critical insights into how environmental and anthropologic factors can be integrated into ASF management strategies to enhance surveillance and control measures. By understanding and adapting to these factors, health authorities can better tailor their interventions, ultimately reducing the impact of ASF outbreaks and protecting both wild and domestic swine populations from this devastating disease. For example, engaging local communities in the surveillance activities can amplify detection efforts, particularly in remote or hard-to-reach areas. Local residents often have unparalleled knowledge of their surrounding environment, which can be invaluable in spotting and reporting potential ASF cases early. This community involvement not only accelerates detection but also fosters a collaborative approach to managing public health threats, which is crucial for effective disease control. This approach is essential for developing more effective, targeted ASF control policies that can mitigate the risk of outbreaks and alleviate the economic and social impacts on affected communities.

## Figures and Tables

**Table 1:** Descriptive statistics of postmortem interval, infected period, and environmental and anthrophonic variables at grid level (*n* = 396).

Variable (unit)	Mean (Stdv.)	Median (min.–max.)	CV	Moran's *I* (*p*-value)
Area-specific PMI (days) (time to detection)	8.25 (12.83)	4 (0–90)	1.56	0.062 (0.079)
Area-specific IP (days)	105.96 (124.49)	55 (12–796)	1.17	0.198 (0.001)
Average elevation (m)	465.67 (239.54)	452.68 (23.38–1105.20)	0.51	0.473 (< 0.001)
Proportion of forests (%)	80.98 (15.87)	84.66 (12.72–99.75)	0.20	0.385 (< 0.001)
Proportion of rice fields (%)	2.08 (13.48)	0 (0–98.77)	6.47	0.075 (0.068)
Proportion of waterbody (%)	1.80 (3.52)	0.7 (0–26.53)	1.95	0.144 (0.006)
Average human density (number of inhabitant/km^2^)	50.52 (110.31)	17.71 (4.15–1052.31)	2.18	0.127 (0.013)
Road density (%)	0.9 (0.85)	0.75 (0–6.01)	0.94	0.149 (< 0.001)
Average cost distance (km)	5.33 (5.01)	3.45 (0.72–30.24)	0.96	0.340 (< 0.001)
Average habitat suitability for wild boars	0.46 (0.11)	0.49 (0–0.60)	0.23	0.701 (< 0.001)
Topological wetness index	8.67 (0.55)	8.52 (7.78–11.19)	0.06	0.456 (< 0.001)

Abbreviations: CV, coefficient of variation; IP, infected period; PMI, postmortem interval; Stdv, standard deviation.

**Table 2:** Posterior marginal distribution of parameters from Bayesian gamma regression of area-specific postmortem interval with covariates on area-specific infected period.

Variables	Mean	95% credible interval (lower, upper)
Area-specific PMI	0.01	0.00^a^, 0.02
The toal number of samples tested for ASF during infected period	0.01	0.00^a^, 0.01
Average habitat suitability for wild boars	1.12	−0.81, 1.24
Standard deviation of residual (*ε*)	0.05	0.00, 0.04
Standard deviation of area-specific PMI in neighboring areas (*u*)	0.52	0.18, 0.87

Abbreviation: PMI, postmortem interval.

^a^Represents a value greater than zero, corresponding to statistical significance.

**Table 3:** Summary of hazard ratio of environmental variables and parameter estimates of baseline hazard function and spatial covariance estimated from Bayesian Cox regression on postmortem interval at area level.

Variables	Median		95% credible interval
Hazard ratio	—	—	—
Low road density (ref = high)	0.11	0.04	0.28
Low average tree cover (ref = high)	0.61	0.27	1.49
Low proportion of rice field (ref = high)	0.78	0.35	1.84
Low proportion of waterbody (ref = high)	4.50	1.61	10.86
Cost distance	0.51	0.21	1.23
Total number of samples collected from wild boars^a^	1.02	0.94	1.11
Parameter estimates	—	—	—
Baseline hazard function parameters	—	—	—
Lambda	1.14	0.171	26.67
Alpha	3.49	2.57	4.55
Spatial covariance parameters	—	—	—
Sigma	3.40	2.43	4.57
Phi	0.13	0.05	0.34

Abbreviation: Ref, reference.

^a^Total number of samples collected from wild boars is equal to the sum of samples collected from wild boars from 100 days before the first sampling date to the last sampling date.

## Data Availability

The data that support the findings of this study are available in the Korea Wild Animal Disease Information System at https://wadis.go.kr/dis/viewASFStat2.do. These data were derived from the following resources available in the public domain: National Wild Animal Disease Control Agency of South Korea, https://wadis.go.kr/main.do. Additionally, the environmental and anthropogenic data used in this study are openly available, as described in Section 2.

## Supporting Information

Supporting Information S1: Detail in method of grid-based partitioning for ASF surveillance. S2: Detail in method of spatial analysis using bivariate local Moran's *I*. S3: Detail in method of survival analysis of carcass detection using spatial Cox regression. Table S1: Summary of environmental and anthropogenic variables affecting ASF spread.
