# Supplementary material for: Impact of Carcass Detection Delays on the Sustained Transmission of African Swine Fever Among Wild Boars
Source: Transbound Emerg Dis. 2025 Jul 28;2025:9889895. doi: 10.1155/tbed/9889895 (PMC12321433; doi:10.1155/tbed/9889895)
Supplement: Supporting Information — S1: Detail in method of grid-based partitioning for ASF surveillance. S2: Detail in method of spatial analysis using bivariate local Moran's I. S3: Detail in method of survival analysis of carcass detection using spatial Cox regression. Table S1: Summary of environmental and anthropogenic variables affecting ASF spread. [file 9889895.f1.docx]

**Supplementary #1.**

Under this assumption, we initially partitioned all Korean territory using a hexagon-shaped grid with a size of about 28.26 km^2^, equivalent to the circle with a 3 km radius, based on the home ranges of wild boars in South Korea in prior studies. Only those grids with ASF-positive wild boar were selected for study since our focus was on detecting ASF-positive carcasses, and PMI could only be measured for a carcass. The grids were first screened for the presence of an ASF-positive carcass, and checks were made to ensure that the detection date of the ASF-positive carcass preceded the dates of hunting and trapping ASF-infected wild boars. If the ASF-positive carcass was identified before identification of ASF from hunting and trapping a wild boar, we calculated the PMI for the first ASF-positive carcass found in each grid as an indicator of how long the carcass persisted before detection and removal by the surveillance system.

Additionally, we calculated the infected period (IP) for each grid, defined as the time from the earliest ASF infection date in wild boars in each grid (the sampling date minus the PMI and 12 days that is the interval from onset of infection to death, as per to the last sampling date of an ASF-positive sample in each grid (figure S1 of the supplementary material). This period is presumed to represent the duration each grid was contaminated with the ASF virus, assuming the ASF-positive carcass in a grid originated from ASF-infected wild boars inhabiting within the same grid.

As shown in the figure, these two indicators, used as target variables or response variable of this study, were named an area-specific PMI and IP respectively, because we estimated them at an area level (i.e., a hexagon-shaped grid with a size of about 28.26 km^2^).

**Supplementary #2.**

We utilized the bivariate local Morans` I statistic to explore the local dispersal effect of delayed detection of ASF-infected carcasses on the extended infection period of ASF in neighboring areas. This spatial statistic is defined by $c*{PMI}_{i}\sum_{j}^{n} w_{ij}IP_{j}$, where c is a constant scaling factor, $PMI_{i}$ represents the PMI at area *i*, and $\sum_{j}^{n} w_{ij}IP_{j}$ denotes the weighted average of IP in the neighboring area *j*. The neighborhood of the given area *i*. was defined as areas sharing a border line with area *i*, and the weight for IP, $w_{ij}$, was equally assigned to neighboring areas *j*. The significance of the bivariate local Morans`I statistic was determined using a p-value threshold of 0.05. This analysis was performed using the GeoDa software version 1.20.

Given the statistical association between area-specific PMI and IP found from this study, we moved forward to build a Bayesian gamma regression model to measure the association between these two variables while adjusting for other covariates, including habitat suitability for wild boars and surveillance intensity. The model also incorporated a spatial random effect term using an intrinsic conditional autoregressive (iCAR) function, designed to explain spatial autocorrelation of residuals. An additional term for spatially independent variation of residuals, assumed to be independent and identically distributed, was included in the model formulation:

$$Area-specific infected period \left( IP \right) \sim Gamma \left( \lambda_{i}, \tau\right)$$

$$E\left( Area-specific IP_{i} \right)=\mu_{i}=\frac{\tau}{\lambda_{i}}$$

$$\log\left( \mu_{i} \right)=\beta_{1}+\beta_{2}\times PMI_{i}+\beta_{3}\times habitat suitabilit{y for wild boar}_{i}+\beta_{4}\times the total number of samples_{i}+u_{i}+\varepsilon_{i}$$

A non-informative prior was assigned to the prior distribution of the parameters, including regression coefficients and both spatial and non-spatial residual terms. The marginal posterior distribution of parameters was estimated using a Markov chain Monte Carlo (MCMC) algorithm, producing three chains with a burn-in of 4,000 iterations, a thinning rate of 10, and totaling 50,000 iterations, as implemented in R2Winbugs version 2.1. The convergence of MCMC chains was assessed through visual inspection of the posterior distributions and computation of the Gelman–Rubin statistic. The Deviance Information Criterion (DIC) was utilized to measure and compare the goodness of fit for the model.

**Supplementary #3.**

Within this survival analysis framework, the hazard at any given time *t* is conceptualized as the probability of an ASF-infected carcass being removed at that time. The hazard function, denoted as $h\left( PMI_{i} \right| \theta)$, where $\theta$ encompasses a set of model parameters, represents the instantaneous rate of carcass removal, computed as $\lim_{\Delta t\to0} \frac{P(t\leq PMI<t+\Delta t|PMI>t,\theta)}{\Delta t}$. Within our Cox regression model, the hazard rate for the removal of ASF-positive wild boar carcasses following their detection is assumed to be proportional to the environmental conditions of the detection area, and it increases exponentially from a baseline hazard function defined by:

$$h\left( PMI_{i};\beta,\omega, \eta,u_{i} \right)=h_{0}\left( PMI_{i};\omega\right) exp(\sum\beta_{k}x_{ki}+u_{i})$$

$$h_{0}\left( PMI_{i};\omega\right)=h_{0}\left( PMI_{i};\alpha,\lambda\right)=\alpha\lambda^{\alpha-1}$$

where $\beta,\omega, \eta$ are the regression coefficients, $\alpha,\lambda$ denote the baseline hazard parameters, and *u* represents the covariance parameters of a spatially continuous latent Gaussian field. The exponential covariance function is utilized to model spatial continuity, denoted as $\sigma^{2}\exp\left( -\frac{d}{\phi} \right), \mathrm{where} \sigma^{2} represents the variance,$*d* the Euclidean distance between ASF-affected areas, and specified by a Weibull distribution model, and $\phi$ the decay rate.

The model’s parameters were estimated through a Bayesian framework utilizing a MCMC sampling algorithm, involving three chains with a burn-in of 4,000 iterations, a thinning rate of 10, and totaling 50,000 iterations, as implemented in R2Winbugs version 2.1. Convergence of these chains was assessed via the Gelman–Rubin statistic, and model fit was evaluated using the DIC.

For Bayesian Cox regression, we transformed continuous explanatory variables into categorical variables. Optimal cutpoints for these classifications were determined using Maximally Selected Rank statistics based on the Log-Rank test, facilitated by the R package survminer version. Additional insights on cutpoints for explanatory variables are detailed in the supplementary material. Proportional hazard assumptions for each variable were verified using scaled Schooenfeld residuals plots, where a centralized pattern around zero indicates that assumptions hold, analyzed using the survival R package version 3.31.

The results from the Cox regression revealed that the exponential regression coefficient, $\exp\left( \boldsymbol{\beta} \right)=\frac{h\left( PMI_{i};\beta,\omega, \eta,u_{i} \right)}{h_{0}\left( PMI_{i};\omega\right)}$, is translated into the relative risk of a unit increase in a variable over baseline hazard, termed as the hazard ratio (HR). Thus, an HR greater than one suggests that conditions conducive to faster carcass removal-implying efficient early detection-are present. Conversely, an HR less than one indicates conditions that may prolong detection times, suggesting delayed detection. We conducted the Bayesian Cox regression employing stepwise selection with the aim of identifying explanatory variables associated with the lowest DIC, using the R software package spatsurv version 1.8.

**Supplementary Table S1.** Data source of environmental and anthropogenic variables

| **Variable (unit)** | **Description** | **Source (year)** |
| --- | --- | --- |
| Elevation (m) | Average elevation of each ASF-positive grid | Global Digital Elevation Model  USGS SRTM (2014) |
| Proportion of forests (%) | Proportion of land area covered by forest within the ASF-positive grid | Land use map  Ministry of Environment, South Korea (2017) |
| Proportion of rice fields (%) | Proportion of land area covered by rice fields within the ASF-positive grid |  |
| Proportion of waterbody (%) | Proportion of land area covered by water body (e.g., lake, river, and stream) within the ASF-positive grid |  |
| Human density  (no. of inhabitant/km^2^) | Average human density of each ASF-positive grid | Worldpop.org  (2019) |
| Road density (%) | Proportion of real-scope roads within the ASF-positive grid | Ministry of the Interior and Safety, South Korea  (2018) |
| Cost distance (m) | Average distance from the residential area, as the function of cost defined as the elevation | Derived from Global Digital Elevation Model USGS SRTM (2014) |
| Topological wetness index | Average level of water accumulation and local drainage as a function of the total catchment area, flow width and slope gradient. | Derived from Global Digital Elevation Model USGS SRTM (2014) |
| Habitat suitability for wild boars (probability) | Average habitat suitability of wild boar each ASF-positive grid | Previous research  (Kim & Park, 2021) |

USGS, United States Geographical Survey; SRTM, Shuttle Radar Topography Mission
